# Does Antenatal Risk Stratification Match Initial and Eventual Model of Care Allocation? A 5‐Year Multi‐Centre Review of Risk Factors and Outcomes

**DOI:** 10.1111/ajo.70058

**Published:** 2025-07-29

**Authors:** James Brown, Serena Yu, Richard De Abreu Lourenco, Monica Zen

**Affiliations:** ^1^ Department of Women's and Newborn Health Westmead Hospital Westmead New South Wales Australia; ^2^ Centre for Health Economics Research and Evaluation (CHERE) University of Technology Sydney Sydney New South Wales Australia

**Keywords:** antenatal care, model of care, perinatal outcomes, pregnancy, risk

## Abstract

**Background:**

Every woman who books into a public hospital for antenatal care in Australia is assessed for risk factors for adverse outcomes. However, no study has examined empirical patterns of risk stratification, subsequent models of care and their relationship to perinatal outcomes.

**Aims:**

This study aims to describe patterns of risk stratification and their intersection with allocated models of care and subsequent perinatal outcomes.

**Materials and Methods:**

This is a multi‐centre retrospective cohort study of all pregnancies booked in and delivered at the three maternity units in western Sydney between 1 January 2018 and 31 December 2022. Women were classified into one of three risk categories (A, B or C) as defined by the Australian College of Midwives' guideline. Variables measured include allocated models of care, maternal and fetal risk factors, birth outcome, and pregnancy morbidity and mortality.

**Results:**

At time of both booking‐in and birth admission, most women were classified as Category C (‘high risk’). During antenatal care, the number of women classified as Category C grew by 70.7% from 21,847 at booking in to 37,290 at birth admission. Between booking‐in and admission for birth, there was an over 25% increase in women allocated to medical models of care during the study period. There was higher perinatal morbidity in women classified as ‘high risk’.

**Conclusions:**

Current antenatal risk stratification methods appear to detect women with a higher chance of adverse perinatal outcomes, but in doing so classify over three quarters of women as ‘high‐risk’. This has important ramifications for model of care, perceived patient risk, and resource allocation.

## 1 Introduction

Every woman who books into a public hospital for antenatal care in Australia is assessed for risk factors for adverse outcomes [1]. These risk factors may be maternal, fetal, and/or obstetric—and may be independent or synergistic [2]. The perceived severity of the identified risk factors then informs the model of care to which a woman will be allocated during her pregnancy, in addition to other considerations. Despite this standardised approach, there is no international consensus about the risk factors for adverse clinical outcomes for pregnancy [3].

Antenatal risk stratification originated in the 1960s as a means of allocating finite care resources to women who needed it most—such pregnancies were labelled ‘high risk’ [4]. Over the intervening decades, these algorithms have expanded and grown into the guidelines we now use [5]. A 2020 systematic review of antenatal risk stratification guidelines identified that most algorithms favour a three‐tier system: low, medium and high risk, which can be functionally interpreted as translating to midwifery care, assisted care and specialised care [6]. However, the relationship between these categories and how women receive antenatal care has not been studied.

Midwives and medical officers in Australian hospitals typically use the consensus guideline developed by the Australian College of Midwives (ACM) [7] for risk classification. Like other stratification algorithms, this guideline triages women into one of three categories—Category A, B, or C—which roughly translate into low, medium, and high risk. In accordance with the guideline, Category A women are appropriate for exclusively midwife‐led care, Category B women are recommended to be consulted by a medical officer when a risk factor is identified (either at booking in or when it emerges in pregnancy), and Category C women are recommended to be referred to a relevant medical practitioner or other health care provider [7]. Women in Category C are not mandated to have obstetrician‐led care, although this may be appropriate depending on the nature of the condition that places them in that category. For example, the presence of pre‐existing Type 1 Diabetes results in classification to Category C, whilst stable hypothyroidism is considered a Category B risk factor.

A woman's risk category is initially assessed and documented by a midwife at booking‐in, but may change during the pregnancy as she is identified to have been exposed to or displays different risk factors, and may lead to a reallocation in her model of care [8, 9]. The guideline is implemented differently between health care facilities, depending on resources and personnel available.

As maternal comorbidities increase [10], and more potential risk factors are researched [11], more women may potentially be classified as Category B and/or C. Classification of a pregnancy in the highest risk category has important implications on the woman, the clinicians and the system itself. Literature indicates that labelling of ‘high risk’ influences how women perceive their own pregnancy and has an impact on decision‐making about pregnancy and childbirth [12, 13, 14]. Similarly, clinicians may be influenced to investigate and intervene in ‘high risk’ pregnancies in situations that may be observed for women in a less serious risk category [15]. For the healthcare system, high risk pregnancies are expected to be cared for through hospital‐based medical models of care where available, which are considered more costly [16].

Therefore, an ideal risk stratification algorithm must balance the countervailing forces of being sufficiently sensitive to identify women who need higher levels of monitoring and care, whilst maintaining the specificity to only label pregnancies ‘high risk’ when such risk of morbidity and mortality truly exists. Beyond this, women's preferences and choices should also be considered in this process.

To date, there has been no Australian study examining patterns of antenatal risk stratification and their relationship to birth outcomes and/or model of care allocation. This study aims to describe patterns of risk classification in a large Australian health district, allocation to model of care and subsequent perinatal outcomes.

## 2 Materials and Methods

This is a multi‐centre retrospective cohort study of data from all pregnancies booked‐in and delivered at three maternity units in Western Sydney between 1 January 2018 and 31 December 2022. This includes a Level 6, Level 5, and Level 3 maternity unit within the same Local Health District (LHD) [17].

### 2.1 Inclusion and Exclusion Criteria

All births at the three hospitals during the study period were included. Women were only excluded from the analysis if they were initially booked in to deliver under the care of a private obstetrician or midwife; however, if they were initially booked in under public care but completed their pregnancy under private care in one of the public hospitals, these records were included. Such cases were included as they still underwent an initial risk stratification and model of care allocation. Multiple pregnancies were included as a single record of antenatal and intrapartum care; however, fetal outcomes are included separately for each delivered baby in a multiple gestation.

### 2.2 Data Collection

Study data were extracted from the eMaternity electronic database, which is the primary repository of routinely collected maternity information within the studied hospitals. Data in eMaternity is contemporaneously collected by clinicians during a pregnancy through history, examination, and investigations. Records that met the inclusion criteria were obtained and de‐identified by JB. Data included demographics, models of care allocation, maternal and fetal risk factors, birth outcome and pregnancy morbidity and mortality—which maintained the default categories as recorded in eMaternity. No free text fields were analysed for this study.

Risk stratification was performed manually from the study data by JB. Women were risk stratified into categories A, B, and C as defined by the Australian College of Midwives' *National Midwifery Guidelines for Consultation and Referral 4th Edition* (2021) (‘ACM guideline’). Risk factors identified in eMaternity were graded individually into the three categories by cross‐referencing each variable with the respective entry in the ACM guideline and allocating the category of A, B or C. Then, each pregnancy was stratified into category A, B or C identifying the highest order risk factor present according to the ACM guideline. For example, the presence of any Category C risk factor would denote that pregnancy as Category C regardless of the presence or volume of Category A or B risk factors. This was performed separately for initial booking in and at the time of birth admission.

Similarly, the allocated model of care was defined at booking‐in and at birth admission, as defined by the categories present in eMaternity. Midwifery models of care included [18]: hospital‐based midwifery, midwifery group practice, midwifery continuity‐of‐care, and community‐based midwifery clinics. Medical models of care included low, medium and high‐risk hospital‐based medical clinics, including maternal‐fetal medicine. GP (General Practitioner) shared‐care included both midwifery and medical GP‐shared care models. ‘Other’ included women who received care through remote area maternity care or who received no antenatal care.

### 2.3 Statistical Analyses

Statistical analyses were performed using STATA Version 18.0 [19]. Student's *t*‐test and the *χ* ^2^ test were used to compare differences in means of continuous and categorical demographic variables between groups, respectively. Differences between groups (e.g., risk categories, or women in different models of care) were considered statistically significant if the *p* value was < 0.05.

### 2.4 Ethics Statement

Ethics approval for the study was obtained from the Western Sydney Local Health District Ethics Committee (HREC reference: 2302‐07), with ratification by the University of Technology Sydney (UTS HREC reference: ETH21‐6090).

## 3 Results

Of 52,231 births over the study period at the three study hospitals between 1 January 2018 and 31 December 2022, data from 48,270 discrete pregnancy episodes were included; 785 duplicate multiple pregnancy records and 3323 women initially booked in to private obstetric or midwifery care were excluded.

Demographics by initially allocated model of care are included in Table 1. There were significant differences between models of care across all demographic variables.

**TABLE 1:** Demographics by initial allocation of model of care.

	Allocated model of care				Total
	Midwifery	Medical	GP shared care	Other	
*N* (%)	31,455 (65.2)	14,982 (31.0)	1603 (3.3)	230 (0.5)	48,270 (100.0)
Maternal age (mean, SD)	30.6 (4.9)	31.9 (5.4)	30.7 (4.6)	28.7 (5.8)	31.0 (5.1)
Gravidity (mean, SD)	2.3 (1.5)	3.1 (2.0)	2.3 (1.4)	3.5 (2.5)	2.6 (1.7)
Parity (mean, SD)	0.9 (1.1)	1.4 (1.4)	0.9 (1.1)	1.9 (2.1)	1.0 (1.2)
Maternal BMI (mean, SD)	24.9 (4.6)	28.3 (7.5)	24.4 (4.4)	27.2	25.9 (5.8)
Born in Australia or New Zealand (*N*, %)	11,160 (35.5)	6249 (41.7)	1248 (77.9)	99 (43.0)	17,895 (37.1)
Birth hospital					
Level 6 unit (*N*, %)	15,166 (48.2)	6780 (45.3)	1082 (67.5)	85 (37.0)	23,113 (47.9)
Level 5 unit (*N*, %)	11,142 (35.4)	7122 (47.5)	242 (15.1)	95 (41.3)	18,601 (38.5)
Level 3 unit (*N*, %)	5147 (16.4)	1080 (7.2)	279 (17.4)	50 (21.7)	6556 (13.6)
Gestation at booking in (weeks, mean, SD)	17.6 (5.2)	19.2 (7.1)	18.1 (5.4)	35.2 (7.0)	18.2 (6.0)

### 3.1 Model of Care Allocations at the Beginning and End of Pregnancy

**FIGURE 1:**
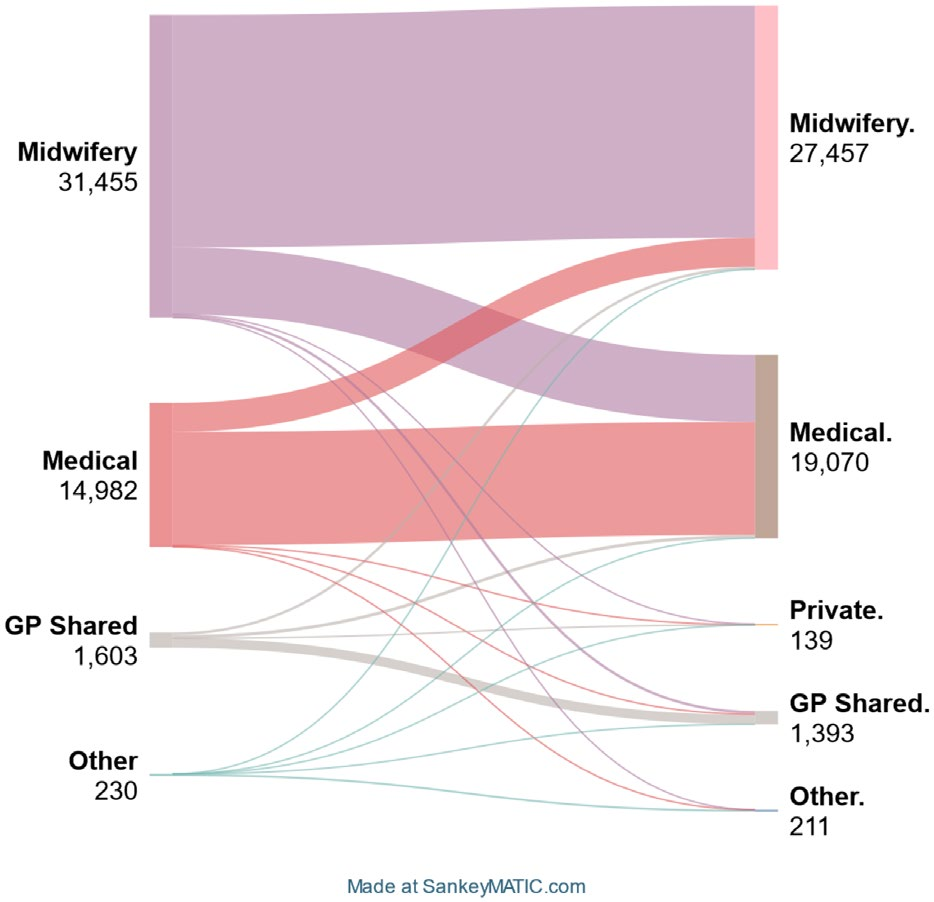
Changes in model of care between booking‐in and birth admission—Sankey Diagram.

Between booking‐in and admission for birth, there was an over 25% increase in women allocated to medical models of care, from 14,982 to 19,070 of the 48,270 pregnancies (Figure 1, Appendix 1). Of the 14,982 women allocated to medical models of care, 918 were allocated to high‐risk maternal‐fetal medical clinics (6.1%); at birth admission, this proportion had fallen to 2.9%. Meanwhile, midwifery and GP‐shared care allocations dropped by 12.7% and 13.1%, respectively. Despite this, most women were allocated to midwifery‐only care at both booking‐in and birth admission.

A small proportion of women (0.3%) elected for private care within the public hospital after originally booking in as a public patient.

### 3.2 Risk Stratification Trends at the Beginning and End of Pregnancy

At time of both booking‐in and birth admission, most women were classified as Category C (‘high risk’) (Table 2). Between booking‐in and birth admission, the number of women classified as Category C grew by 70.7% to 37,290, representing over three quarters of all women in the study population. Approximately one in ten women were considered ‘low risk’ at the time of birth admission. The flow between category groups is described visually in Appendix 2.

**TABLE 2:** ACM risk category prevalence at booking‐in and at birth.

ACM risk category	At booking‐in (*N*)	At birth admission	Change (%)
A	10,408 (21.6%)	5463 (11.3)	−47.5
B	16,015 (33.2)	5517 (11.4)	−65.6
C	21,847 (45.3)	37,290 (77.3)	+70.7
Total	48,270 (100.0)	48,270 (100.0)	

At time of booking‐in, 28.1% of women allocated to a midwifery model of care were Category A, with 35.7% Category B and 36.3% Category C (Appendix 3). By birth admission, most women in midwifery models of care were Category C (69.7%), whereas the Category A proportion had shrunk to 16.8%.

A similar pattern is observed in women allocated to medical models of care. At booking‐in, 64.9% were Category C, which grew to 88.9% by birth admission. Women booked into GP‐shared care were evenly distributed across the risk categories at booking‐in but followed a similar pattern to midwifery and medical models of care by birth admission.

### 3.3 Relationship Between Risk Category, Model of Care and Adverse Outcomes

There are significant differences in neonatal outcomes by allocated model of care at booking‐in and admission (Appendices 4 and 5). This includes a greater proportion of neonatal deaths and stillbirths in women in a medical model of care—as well as earlier gestation at delivery, lower birthweight and more frequent admission to NICU.

Women booked in to midwifery clinics had a lower rate of caesarean section than women booked in to medical clinics (26.6% vs. 43.8%), they had a higher rate of forceps and vacuum delivery (4.9%, 6.0%) compared to the medical cohort (3.0%, 3.8%).

The worse outcomes identified in the Other model of care group were due to most of those women not receiving antenatal care.

ACM risk category at booking‐in was not associated with a statistically significant difference in stillbirth and neonatal death (Appendix 6), but this link strengthened for ACM risk category at time of birth admission (Table 3). When considering ACM risk category at birth admission, there was little difference in outcome parameters between Category A and Category B, beyond a higher prevalence of large‐for‐gestational‐age (7.3% vs. 9.5%). However, women in Category C at birth admission experienced adverse outcomes more frequently, includingpre‐term birth (9.9%), fetal weight below the 5th centile (4.8%), and admission to special care nursery or neonatal intensive care unit (18.3%).

**TABLE 3:** Neonatal outcomes by ACM risk category at birth admission.

	ACM risk category at birth admission			*p*
	A	B	C	
*N* (%)	5463 (11.2%)	5517 (11.3)	37,928 (77.5)	
Neonatal outcome				< 0.001
Neonatal death	7 (0.1)	6 (0.1)	128 (0.3)	
Stillbirth	22 (0.4)	26 (0.5)	227 (0.6)	
Gestation at delivery (mean)	39.6	39.3	38.6	< 0.001
37 weeks or greater	5395 (98.8)	5424 (98.3)	34,179 (90.1)	
34–36 + 6 weeks	53 (1.0)	60 (1.1)	2412 (6.4)	
30–33 + 6 weeks	7 (0.1)	11 (0.2)	677 (1.8)	
Less than 30 weeks	8 (0.1)	22 (0.4)	660 (1.7)	
Birthweight (kilograms, mean)	3.35	3.37	3.23	< 0.001
< 5 Centile	121 (2.2%)	127 (2.3%)	1803 (4.8%)	
5–10 centile	191 (3.5%)	176 (3.2%)	1403 (3.7%)	
> 10 to < 95 centile	4752 (87.0%)	4688 (85.0%)	30,954 (81.6%)	
> 95 centile	399 (7.3%)	526 (9.5%)	3768 (9.9%)	
Apgar < 7 at 5 min	52 (1.0%)	79 (1.4%)	1102 (2.9%)	< 0.001
Admission to NICU or special care nursery	503 (9.2%)	498 (9.0%)	6928 (18.3%)	< 0.001

## 4 Discussion

This is the first study to examine the relationship between antenatal risk stratification, allocated model of care, and adverse perinatal outcomes.

The original mandate of risk stratification algorithms was to identify the minority of women who would benefit from intensive multidisciplinary care. These findings show that the output of these algorithms have deviated from this goal and reveal that over three quarters of women are now classified as ‘high risk’ by birth admission. Over the last decades, risk stratification algorithms appear to have expanded to include any number of emerging factors that may increase the likelihood of an adverse perinatal outcome, without attention to how this affects appropriate care pathways. Moreover, the current risk stratification methodology does not wholly inform initial and eventual model of care allocation—with considerable diversity in risk categories observed within all models of care. This has important ramifications for available models of care, perceived patient risk, and resource allocation.

The observed mismatch between risk classification and model of care allocation is striking. Many high and medium risk women are seen in traditionally low risk models of care; and in contrast, a low‐risk cohort was initially allocated to medical models of care. At booking‐in, one in three women receiving midwifery‐only care are Category C, rising to almost three quarters by birth admission. The most likely reason for this pattern is that risk stratification is only a minor consideration when clinicians allocate women to models of care. At the point of risk stratification, clinicians consider both clinical guidelines and clinical judgement to allocate the model of care. The augmentation with clinical judgement allows for more individualised risk allocation that permits flexibility. This assumption is strengthened by the similar performance of risk category and model of care in identifying stillbirth and neonatal death in this study.

Consequently, the current method of risk stratification is not serving its intended purpose. Triage algorithms are designed to identify women at risk and standardise the care they receive to ensure their needs are met. The data show that the current risk criteria do not achieve this. Without a transparent and partially predictable risk allocation system, there is no way for maternity services to allocate resources efficiently and for women to be informed as to how their care will be delivered.

This raises an important ethical question: Are we satisfied with labelling three quarters of all pregnant women as ‘high risk’? It seems paradoxical for most of a population to be described as having a disproportionately high level of risk; outliers cannot be a majority. Instead, it suggests misallocation of the risk median. In this study most women would be considered ‘high risk’, whilst only one in ten will still be ‘low risk’ at birth admission. This stands in opposition to the common belief, and policy‐supported position, that birth is a natural process that should be unhindered by intervention unless justified by specific concerns [20]. It appears that currently the risk stratification methodology prioritises sensitivity to risk over specificity of risk to achieve its goals.

This hyper‐sensitive triage does not necessarily result in better outcomes. Whilst there are statistically significant differences in adverse outcomes between the risk categories at time of birth admission, the relative difference in prevalence between the groups is far more subtle than the labels of low, medium and high risk would indicate. Small for gestational age, stillbirth and neonatal death still occur in the low and medium risk cohorts at clinically significant frequencies. It is not possible to isolate the effect of model of care on outcomes, nor is it feasible to further expand the high‐risk category to encompass more women.

More practically, we know that the label ‘high risk’ itself is not benign and influences how the woman and her care providers view and act towards the pregnancy. Therefore, the labelling process could be considered a consequential intervention—one that may affect the real outcomes of the pregnancy. For example, a woman or her care provider may be more proactive in seeking an elective caesarean section or induction at an earlier gestation due to concerns about having a ‘high‐risk’ pregnancy.

Conversely, this study identified that adverse outcomes occur at a clinically significant rate in pregnancies where women were identified as low or medium risk, where women and staff may have a higher threshold to act on early signs of concern.

The COVID‐19 pandemic occurred during the study period, and the 2021 ACM guideline considered COVID‐19 infection during pregnancy a Category C risk factor. However, after removing COVID‐19 in pregnancy as a risk factor, the number of women considered Category C at birth admission dropped from 37,290 (77.3%) to 36,829 (76.3%) meaning this factor only had a subtle influence on the patterns described in this study.

There are limitations to this study. It is unable to extrapolate the effect of risk stratification or model of care on the occurrence of adverse outcomes, because the risk groups are inherently different and therefore cannot be controlled for one another. For example, we are unable to determine whether adverse outcomes in the low‐risk groups could have been predicted by a different risk stratification model, nor can we determine whether the adverse outcomes would have been prevented if allocated to a different model of care.

The study was also unable to define the timepoints in pregnancy when women moved risk category due to only two data timepoints being available. Also, the observed statistically significant differences in demographics between the sites was an expected finding given the different capacity for patient complexity across the hospitals in the district.

The analysis also relies on the quality of data in the eMaternity database. This data is typically considered to be a reliable source but may be limited by the accuracy of user input [21].

In conclusion, this is the first study of its kind and reveals important insights into the relationship between risk stratification, models of care and patterns of adverse outcomes. It raises important questions about what a ‘high risk’ label truly means, who should receive this label, and the implications for how and where pregnancy is cared for. The findings advocate for urgent work on a more nuanced antenatal risk stratification method.

## Conflicts of Interest

The authors declare no conflicts of interest.

## Appendix 1 Changes in Model of Care Between Booking‐In and Birth Admission

**Table:**

	Model of care allocation		Change (%)
	At booking‐in (*N*)	At birth (*N*)	
Midwifery	31,455 (65.2%)	27,457 (56.9)	−12.7
Medical^a^	14,982 (31.0)	19,070 (39.5)	+27.9
GP shared care	1603 (3.3)	1393 (2.9)	−13.1
Other	230 (0.5)	211 (0.4)	−8.3
Private		139 (0.3)	
Total	48,270 (100.0)	48,270 (100.0)	

^a^ 918 were allocated to high‐risk maternal‐fetal medicine clinics at booking‐in, whilst 553 were allocated by birth admission.

## Appendix 3 ACM Risk Category Prevalence at Booking‐In and at Birth, by Model of Care

**Table:**

	At booking‐in (*N*)	At birth admission (*N*)	Change (%)
Midwifery	31,455 (65.2%)	26,803 (56.4)	−14.8
Category A	8819 (28.1)	4508 (16.8)	
Category B	11,223 (35.7)	3623 (13.5)	
Category C	11,413 (36.3)	18,672 (69.7)	
Medical	14,982 (31.1)	18,961 (39.9)	+26.6
Category A	1069 (7.1)	556 (2.9)	
Category B	4196 (28.0)	1543 (8.1)	
Category C	9717 (64.9)	16,862 (88.9)	
GP shared care	1603 (3.3)	1382 (2.9)	−13.8
Category A	498 (31.1)	262 (19.0)	
Category B	496 (30.9)	190 (13.7)	
Category C	609 (38.0)	930 (67.3)	
Other	230 (0.5)	211 (0.4)	−8.3
Category A	22 (9.6)	4 (1.9)	
Category B	100 (43.5)	54 (25.6)	
Category C	108 (47.0)	153 (72.5)	
Private		139 (0.3)	
Category A		7	
Category B		9	
Category C		123	
Total	48,270 (100.0)	47,496 (100.0)	

## Appendix 4 Neonatal Outcomes by Model of Care Allocation at Booking in

**Table:**

	Model of care allocated at booking‐in				*p*
	Midwifery (*N*)	Medical	GP shared care	Other	
*N* (%)	31,473 (64.4%)	15,599 (31.9)	1604 (3.3)	232 (0.5)	
Neonatal outcome					< 0.001
Neonatal death	59 (0.2)	76 (0.5)	4 (0.2)	2 (0.9)	
Stillbirth	138 (0.4)	125 (0.8)	4 (0.2)	8 (3.4)	
Gestation at delivery (mean)	39.2	38.0	39.2	36.5	< 0.001
37 weeks or greater	30,026 (95.4)	13,280 (85.1)	1531 (95.4)	161 (69.4)	
34–36 + 6 weeks	999 (3.2)	1447 (9.3)	50 (3.1)	29 (12.5)	
30–33 + 6 weeks	214 (0.7)	454 (2.9)	13 (0.8)	14 (6.0)	
Less than 30 weeks	234 (0.7)	418 (2.7)	10 (0.6)	28 (12.1)	
Birthweight (kilograms, mean)	3.32	3.13	3.31	2.93	< 0.001
< 5 Centile (for gestational age)	1010 (3.2)	955 (6.1)	53 (3.3)	33 (14.2)	< 0.001
5–10 centile	1099 (3.5)	604 (3.9)	61 (3.8)	6 (2.6)	
> 10 to < 95 centile	26,495 (84.2)	12,406 (79.5)	1337 (83.4)	156 (67.2)	
> 95 centile	2869 (9.1)	1634 (10.5)	153 (9.5)	37 (15.9)	
Apgar < 7 at 5 min	519 (1.6)	658 (4.2)	24 (1.5)	32 (13.8)	< 0.001
Admission to NICU or special care nursery	3879 (12.4)	3777 (24.4)	199 (12.5)	74 (32.3)	< 0.001

## Appendix 5 Neonatal Outcomes by Model of Care Allocation at Birth Admission

**Table:**

	Model of care allocated at birth admission					*p*
	Midwifery (*N*)	Medical	GP shared care	Other	Private	
*N* (%)	26,818 (55.7%)	19,571 (40.7)	1384 (2.9)	213 (0.4)	147 (0.3)	
Neonatal outcome						< 0.001
Neonatal death	43 (0.2)	94 (0.5)	0 (0.0)	2 (0.9)	1 (0.7)	
Stillbirth	94 (0.4)	166 (0.8)	3 (0.2)	8 (3.8)	2 (1.4)	
Gestation at delivery (mean)	39.3	38.1	39.2	36.5	37.5	< 0.001
37 weeks or greater	25,887 (96.5)	16,772 (85.7)	1327 (95.9)	147 (69.0)	115 (78.2)	
34‐36 + 6 weeks	693 (2.6)	1,740 (8.9)	33 (2.4)	27 (12.7)	11 (7.5)	
30‐33 + 6 weeks	123 (0.5)	532 (2.7)	15 (1.1)	11 (5.2)	13 (8.8)	
Less than 30 weeks	115 (0.4)	527 (2.7)	9 (0.7)	28 (13.1)	8 (5.4)	
Birthweight (kilograms, mean)	3.36	3.13	3.32	2.93	2.93	< 0.001
<5 Centile (for gestational age)	696 (2.6)	1,242 (6.3)	48 (3.5)	32 (15.0)	9 (6.1)	< 0.001
5‐10 centile	871 (3.2)	814 (4.2)	39 (2.8)	5 (2.3)	11 (7.5)	
>10 to < 95 centile	22,773 (84.9)	15,549 (79.4)	1167 (84.3)	139 (65.3)	117 (79.6)	
> 95 centile	2,478 (9.2)	1966 (10.0)	130 (9.4)	37 (17.4)	10 (6.8)	
Apgar < 7 at 5 min	360 (1.3)	803 (4.1)	19 (1.4)	31 (14.6)	10 (6.8)	< 0.001
Admission to NICU or special care nursery	2953 (11.0)	4605 (23.7)	177 (12.8)	67 (31.9)	36 (25.0)	< 0.001

## Appendix 6 Neonatal Outcomes by ACM Risk Category at Booking‐In

**Table:**

	ACM risk category at booking‐in			*p*
	A	B	C	
*N* (%)	10,534 (21.5%)	16,222 (33.2)	22,152 (45.3)	
Neonatal outcome				0.114
Neonatal death	21 (0.2)	41 (0.3)	79 (0.4)	
Stillbirth	58 (0.6)	95 (0.6)	122 (0.6)	
Gestation at delivery (mean)	39.1	38.9	38.6	< 0.001
37 weeks or greater	9919 (94.2)	15,012 (92.5)	20,067 (90.6)	
34–36 + 6 weeks	431 (4.1)	759 (4.7)	1335 (6.0)	
30–33 + 6 weeks	101 (1.0)	222 (1.4)	372 (1.7)	
Less than 30 weeks	83 (0.8)	229 (1.4)	378 (1.7)	
Birthweight (kilograms, mean)	3.26	3.28	3.25	< 0.001
< 5 Centile	438 (4.2)	623 (3.8)	990 (4.5)	
5–10 centile	445 (4.2)	576 (3.6)	749 (3.4)	
> 10 to < 95 centile	8870 (84.2)	13,433 (82.8)	18,091 (81.7)	
> 95 centile	781 (7.4)	1590 (9.8)	2322 (10.5)	
Apgar < 7 at 5 min	191 (1.8)	402 (2.5)	640 (2.9)	< 0.001
Admission to NICU or special care nursery	1517 (14.5)	2426 (15.0)	3986 (18.1)	< 0.001
